# Feasibility of Left Bundle Branch Area Pacing Combined with Atrioventricular Node Ablation in Atrial Fibrillation Patients with Heart Failure

**DOI:** 10.3390/jcdd9100338

**Published:** 2022-10-05

**Authors:** Qi-Qi Jin, Cheng Zheng, Yao-Ji Wang, Jia-Xuan Lin, Dao-Zhu Wu, Jia-Feng Lin, Xue-Qiang Guan

**Affiliations:** Department of Cardiology, The Second Affiliated Hospital and Yuying Children’s Hospital of Wenzhou Medical University, Xueyuan Road No. 109, Wenzhou 325027, China

**Keywords:** left bundle branch area pacing, left ventricular septal pacing, atrioventricular node ablation, atrial fibrillation, heart failure

## Abstract

Background: Pacemaker implantation combined with atrioventricular node ablation (AVNA) could be a practical choice for atrial fibrillation (AF) patients with heart failure (HF). Left bundle branch area pacing (LBBaP) has been widely reported. Objectives: To explore the safety and efficacy of LBBaP combined with AVNA in AF patients with HF. Methods and results: Fifty-six AF patients with HF attempted LBBaP and AVNA from January 2019 to December 2020. Standard LBBaP was achieved in forty-six patients, and another ten received left ventricular septal pacing (LVSP). The cardiac function indexes and pacemaker parameters were evaluated at baseline, and we conducted a 1-month and 1-year follow-up. Result: At the time of implantation and 1-month and 1-year follow-up, QRS duration of LVSP group was longer than that of LBBaP group. The pacemaker parameters remained stable in both the LBBaP and LVSP groups. At 1-month and 1-year follow-up after LBBaP and AVNA, left ventricular ejection fraction, left ventricular end-diastolic diameter, and NYHA classification continued to improve. Baseline left ventricular ejection fraction and QRS duration change at implantation can predict the magnitude of improvement of left ventricular ejection fraction at 1-year after LBBaP. Baseline right atrial left-right diameter, the degree of tricuspid regurgitation, and interventricular septum thickness may be the factors affecting the success of LBBaP. Conclusion: LBBaP combined with AVNA is safe and effective for patients with AF and HF. Baseline right atrial left-right diameter, the degree of tricuspid regurgitation, and interventricular septum thickness may be the factors affecting the success of LBBaP.

## 1. Introduction

Atrial fibrillation (AF) and heart failure (HF) frequently coexist and can promote each other. In patients with AF who are intolerant or unresponsive to intensive rate and rhythm control therapy, atrioventricular node ablation (AVNA) combined with pacemaker implantation for rate control can be used as an approach of last resort [1].

Long-term right ventricular apical pacing (RVAP) leads to electrical and mechanical dyssynchrony, associated with an increased incidence rate of AF, HF, and pacemaker-induced cardiomyopathy [2,3]. Cardiac resynchronization therapy (CRT) is another indication for the treatment of heart failure. Previous studies have shown that AVNA and CRT significantly improve life quality and overall survival in patients with HF and AF. However, 30–40% of patients who receive CRT therapy do not show significant clinical improvement [4,5].

His-Purkinje system pacing has been used as a physiological pacing strategy. Research shows that AVNA and His bundle pacing (HBP) can improve cardiac function and reduce diuretics use in patients with AF and HF. Nevertheless, HBP has some limitations, including higher pacing thresholds, lower R wave amplitude, and the difficulty of lead implantation, which limited the clinical applications of HBP [6,7,8]. Left bundle branch area pacing (LBBaP) is a novel strategy for CRT which was first recommended by Huang et al. in 2017 [9]. Subsequently, several studies demonstrated the safety, feasibility, and application in patients undergoing LBBaP. LBBaP can overcome some of the clinical limitations of HBP and might provide a more physiological pacing approach than traditional RVAP and a more stable approach than HBP [9,10,11,12]. The present study aimed to explore the safety and efficacy of LBBaP combined with AVNA in patients with AF and HF.

## 2. Methods

### 2.1. Research Object

The present study was a single-center retrospective study, and included fifty-six consecutive AF patients with symptomatic HF who attempted LBBaP and AVNA from January 2019 to December 2020. Studies and data collection were performed according to protocols approved by the Ethics Committee of the Second Affiliated Hospital of Wenzhou Medical University. All patients provided written informed consent. Echocardiogram reports and Pacemaker parameters were available for all patients.

Inclusion criteria were as follows: (1) all patients had New York Heart Association (NYHA) classification class II to IV HF symptoms despite optimal medical therapy; (2) age > 60; (3) brain natriuretic peptide (BNP) increased; (4) transthoracic echocardiography demonstrated abnormalities in cardiac structure and/or function. (5) long-lasting persistent or permanent AF, and heart rate was uncontrolled by medical therapy; or (6) AF that failed several catheter ablations, or intolerance to ablation.

Exclusion criteria were as follows: (1) patients with poor general condition, unable to tolerate surgery; (2) patients with severe heart (severe congenital heart disease, e.g., tetralogy of Fallot, Eisenmenger syndrome), liver (Child-Pugh C grade, e.g., hepatic encephalopathy), kidney (patients with chronic kidney disease stage five who refuse hemodialysis), and other severe dysfunction (e.g., infection, hematological disease, mental illness); (3) patients with serious coagulation disorders; (4) patients with a life expectancy period < 1-year; (5) patients with contrast agent allergy.

### 2.2. Implantation Procedure

#### 2.2.1. Left Bundle Branch Area Pacing

LBBaP was performed using the Select Secure lead (model 3830, Medtronic, Minneapolis, MN, USA). In the right anterior 30° oblique projection, the lead was delivered through the fixed curve sheath (C315His, Medtronic, Minneapolis, MN, USA). The sheath and lead tip were first advanced to the anterior lower site of the His-bundle position and subsequently rotated in a counterclockwise fashion to place the lead tip in a perpendicular orientation to the IVS. At this location, the paced QRS morphology usually displayed a “W” pattern with a midnotch in lead V1 [13].

Then the tip of lead was screwed clockwise until it penetrated deep into the interventricular septum. The QRS changed from left bundle branch block (LBBB) pattern to right bundle branch block (RBBB) pattern [8,14].

The evidence of LBB capture: during unipolar-tip pacing, right bundle branch configuration was observed in addition to one or more of the following findings: 1. recording of a LBB potential, 2. transition from nonselective to selective LBB capture, 3. Stim-LVAT that shortens abruptly with increasing output and remains shortest and constant at high- and low-output pacing [8,14]. Successful LBBaP is defined as having the evidence of LBB capture. If successful LBBaP could not be achieved after five attempts of lead positioning, the lead was then placed in the mid-LV septum to achieve a relatively narrow QRS, which was named left ventricular septal pacing (LVSP) [10].

During the procedure, the paced 12-lead electrocardiogram (ECG), intracavitary electrogram (IECG), pacemaker parameters, pacing stimulus to left ventricular activation time (Stim-LVAT, defined as the duration from pacing spike to the R wave peak of a QRS complex at precordial leads V5 and V6), and the paced QRS duration (the duration from the pacing artifact to the QRS end during unipolar pacing) were monitored.

#### 2.2.2. Atrioventricular Node Ablation

**Figure 1:**
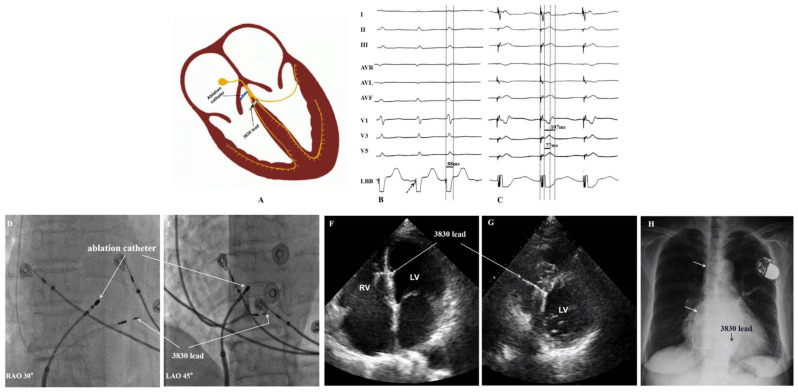
Left bundle branch area pacing. (**A**): Diagram of the LBBaP and AVNA. (**B**): LBB potential recorded from the 3830 lead (dash arrow). Baseline QRSd of 88 ms. (**C**): The 3830 lead implanted in LBB area successfully; unipolar LBBaP pacing at pacing at 1 V/0.4 ms resulted in LBB capture with QRSd of 107 ms and Stim-LVAT of 77 ms. (**D**,**E**): Fluoroscopic imaging of LBBaP lead implantation ((**D**): RAO 30-deegree fluoroscopic views and (**E**): LAO 45-degree fluoroscopic view). (**F**,**G**): Echo images demonstrating the location of the LBBaP lead in the interventricular septum ((**F**): apical 4-chamber view and (**G**): left parasternal short axis transection view). (**H**): Radiograph of chest after LBBaP and AVN ablation (posteroanterior view). LBB = left bundle branch, LBBaP = left bundle branch area pacing, AVNA = atrioventricular node ablation, RAO = right anterior oblique, LAO = left anterior oblique, RV = right ventricle, LV = left ventricle, Stim-LVAT = pacing stimulus to left ventricular activation time, Echo = echocardiography.

AVNA was performed under fluoroscopic guidance. After LBBaP was established, an 8.5 F sheath was inserted through the femoral vein to the right atrium, and a 4 mm irrigated ablation catheter was delivered through the sheath and positioned in the region of the compact atrioventricular node. When the tip of the catheter recorded a near-field Hisian potential, ablation energy was released with a flow rate of 15–30 mL/min, preset power of 35W, and temperature of 43 °C. Desired success of AVNA was evidenced with complete atrioventricular block (AVB) without change in LBBaP parameters (Figure 1) [6].

### 2.3. Data Collection and Postprocedural Follow-Up

Clinical and procedural data were obtained from medical records. Baseline patient characteristics, including the demographic characteristics, patient history, medication use, and NYHA classification, were recorded. The baseline mean heart rate was measured using dynamic electrocardiogram (Holter). Furthermore, 12-lead ECG and echocardiography (Echo) were routinely performed before the procedure. Echo parameters, including left ventricular ejection fraction (LVEF), left ventricular end-diastolic diameter (LVEDd), left atrial anterior-posterior diameter (LAAPD), right atrial left-right diameter (RALRD), right atrial superior-inferior diameter (RASID), right ventricular left-right diameter (RVLRD), and interventricular septum thickness (IVST) were obtained. The severity of mitral regurgitation (MR) and tricuspid regurgitation (TR) was assessed according to current guidelines, defined as follows: 0-None; 1-Mild; 2-Moderate; and 3-Severe [15].

All patients were followed up in the outpatient department. Routine Echo and pacemaker data were recorded at 1-month and 1-year follow-up. Procedure-related complications included capture threshold increase of 1 V, loss of capture, lead septal perforation, lead dislodgement, and infection. Clinical outcomes included mortality and HF hospitalization.

### 2.4. Statistical Analysis

Continuous variables were described as mean ± standard deviations (SD) or median with interquartile range (IQR, 25–75%). Student’s t-tests were used for parameters that were normally distributed (paired-sample t-test and independent-sample t-test were used for intra- and intergroup comparisons, respectively). Wilcoxon signed-rank tests were used for within-group comparisons for parameters with skewed distribution, and Mann–Whitney U-tests were used for between-group comparisons. Data comparison at different time-points was conducted by repeated-measures analysis of variance. Pairwise comparisons were performed post hoc to identify which groups were different. Categorical data were expressed as numbers (%). Qualitative data were compared using Chi-squared or Fisher’s exact tests. Multiple linear regression models were constructed to predict the prognosis of operation. Statistical analyses were conducted using SPSS version 23.0 (IBM, Armonk, New York, NY, USA). All tests were two-sided. A *p*-value < 0.05 was considered statistically significant.

## 3. Results

### 3.1. Baseline Characteristics

Fifty-six consecutive AF patients with symptomatic HF attempted LBBaP and AVNA from January 2019 to December 2020. Forty-six patients were successfully treated with permanent LBBaP as LBBaP group, and ten patients failed at five attempts of lead positioning and finally received permanent LVSP as LVSP group. Baseline characteristics of the study population are shown in Table 1. There was no significant difference in baseline characteristics such as age and sex among the patients of these two groups. However, there were more patients with cardiomyopathy in LVSP group (*p* = 0.003).

### 3.2. Baseline and Pacing Heart Rates, QRSd, and Stim-LVAT 

There were no significant differences in baseline heart rate, pacing heart rate and baseline QRSd between LBBaP and LVSP groups (*p* = 0.743, *p* = 1.000, and *p* = 0.092). At the time of implantation and 1-month and 1-year follow-up, QRSd of the two groups were longer than that before operation (all *p* < 0.05). QRSd and stim-LVAT of LVSP group were longer than that of LBBaP group during the same period (all *p* < 0.001) (Table 2).

### 3.3. Pacemaker Parameters and Complications

The pacemaker parameters remained stable at 1-month and 1-year follow-up after permanent LBBaP and AVNA. The pacing threshold and impedance were lower than those at implantation, and the impedance decreased significantly with statistical difference (*p* < 0.001) (which may be related to the gradual fixation of pacing lead with surrounding myocardial tissue and making good contact with myocardium). No patient demonstrated an increase in the pacing threshold above 1V (compared to baseline). The median ventricular pacing rates were 99.0% and 99.8%, respectively (Table 3). Incomplete RBBB occurred in two cases and returned to normal before discharge. Pacemaker pocket hematoma occurred in one case, in which the bleeding was stopped with a compression bandage and the hematoma was absorbed spontaneously. No severe complications such as pacemaker pocket infection, dislocation of the pacemaker lead, premature exhaustion of pacemaker batteries, or pacemaker-related cardiac perforation occurred at implantation or during the follow-up period. After permanent LVSP, the pacemaker parameters also remained stable over time. No serious complications were observed during or after the operation (Table 3).

### 3.4. Echo Parameters and NYHA Classification 

A series of Echo parameters largely improved after LBBaP and AVNA: 1) the improvements in LVEF and LVEDd were present at 1-month of LBBaP and more improvements were observed at 1-year follow-up ((53.0 ± 11.9 vs. 56.7 ± 11.1 vs. 60.4 ± 8.8)%, *p* < 0.05 for all pairwise comparisons; (49.5 ± 5.8 vs. 48.4 ± 5.7 vs. 46.3 ± 5.6) mm, *p* < 0.05 for all pairwise comparisons). Decreases or improvements were also observed in the LAAPD, RALRD, and the degree of MR at 1-year follow-up ((47.8 ± 7.3 vs. 45.8 ± 3.8) mm, *p* = 0.001 and (45.1 ± 6.6 vs. 43.6 ± 6.9) mm, *p* = 0.031 and *p* = 0.025). There was no significant change in the degree of TR, RVLRD, and IVST (all *p* > 0.05). However, the changes of Echo parameters did not reach statistical differences in the LVSP group (Table 4). There were no significant differences in the improvement of Echo parameters between LBBaP and LVSP groups during the follow-up time (all *p* > 0.05), while greater numerical improvements were observed in LVEF, LVEDd, and LAAPD at the LBBaP group (Table 5).

At 1-month and 1-year follow-up after LBBaP and AVNA, NYHA classification continued to improve (*p* < 0.001 for all pairwise comparisons). In the LVSP group, the improvement of NYHA classification reached statistical difference at 1-year after operation (*p* = 0.025) (Table 4). A more significant improvement in NYHA classification was observed in LBBaP group at 1-year follow-up when compared with the LVSP group (*p* = 0.021) (Table 5).

**Figure 2:**
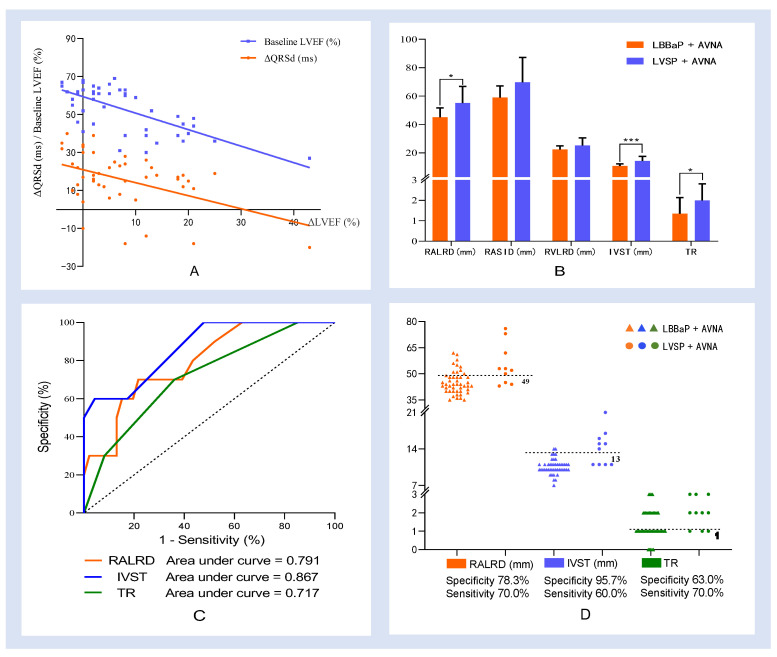
(**A**) Baseline LVEF and ΔQRSd predicted the magnitude of improvement of LVEF at 1 year after LBBaP and AVNA. The multiple linear regression equation is “ΔLVEF = 44.295-0.463 * baseline LVEF-0.182 * ΔQRSd”, indicating that the lower the baseline LVEF, the smaller the QRSd broadening, or the more obvious the QRSd shortening at implantation, the more significant the improvement of LVEF at 1-year follow-up. (**B**) Comparison of baseline Echo parameters between LBBaP and LVSP groups. There were statistical differences in RALRD, IVST, and the degree of TR between the two groups. (**C**,**D**) Scatter plots and ROC curve. The cut of values of RALRD, IVST, and the degree of TR were 49mm, 13mm, and 1(mild regurgitation), respectively. Additionally, the AUC values were 0.791, 0.867 and 0.717, respectively. When the RALRD > 49 mm, IVST > 13 mm, and the degree of TR were moderate or severe, LBBaP was more likely to fail. LVEF = left ventricular ejection fraction, IVST = interventricular septum thickness, TR = tricuspid regurgitation, RALRD = right atrial left-right diameter, LBBaP = left bundle branch area pacing, LVSP = left ventricular septal pacing, AVNA = atrioventricular node ablation, Echo = echocardiography, ΔLVEF = the magnitude of improvement of LVEF at 1-year after LBBaP and AVNA, ΔQRSd = QRS duration change at implantation, AUC = area under ROC curve, ROC = receiver operating characteristic curve, AUC = area under the curve. *** *p* < 0.001, * *p* < 0.05; the degree of tricuspid regurgitation defined as 0-None, 1-Mild, 2-Moderate; and 3-Severe.

The magnitude of improvement of LVEF at 1-year after LBBaP and AVNA (recorded as ΔLVEF) was taken as the prognostic indicator. Multiple linear regression models were constructed to predict the prognosis of operation. Baseline variables that were considered clinically relevant and candidate variables with *p*-values < 0.1 on univariate analysis were included in the multivariable model. The final variables included baseline LVEF, stim-LVAT, postoperative change in ventricular rate (record as ΔHR), and QRSd change at implantation (record as ΔQRSd). The established regression model was statistically significant, F = 12.293, *p* < 0.001, and adjusted R^2^ = 0.501. Among the four variables included, baseline LVEF and ΔQRSd were statistically significant (*p* < 0.001 and *p* = 0.019). The multiple linear regression equation is “ΔLVEF = 44.295-0.463 * baseline LVEF-0.182 * ΔQRSd”, indicating that the lower the baseline LVEF, the smaller the QRSd broadening, or the more obvious the QRSd shortening at implantation, the more significant the improvement of LVEF at 1-year follow-up (Figure 2A).

We compared the baseline indicators in the LBBaP and LVSP groups to analyze the factors that may affect the success of LBBaP. We found that patients who received LVSP after a failed attempt of LBBaP had larger RALRD, more severe TR, and thicker IVST ((45.1 ± 6.6 vs. 55.1 ± 11.0) mm, *p* = 0.010; 1.0 (1.0, 2.0) vs. 2.0(1.0, 3.0), *p* = 0.028; 10.0 (10.0, 11.0) vs. 14.5 (11.0, 16.3) mm, *p* < 0.001) (Table 4, Figure 2B), suggesting that baseline RALRD, the degree of TR, and IVST may be the factors affecting the success of LBBaP. Scatter plots and ROC curve are shown in Figure 2C,D. The cut of values of RALRD, IVST, and the degree of TR were 49 mm, 13 mm, and 1(mild regurgitation), respectively. Additionally, the area under the curve (AUC) values were 0.791, 0.867 and 0.717, respectively. When the RALRD > 49 mm, IVST > 13 mm, and the degree of TR were moderate or severe, LBBaP was more likely to fail, and the sensitivity, specificity, positive predictive value, and negative predictive value were 70.0%, 78.3%, 41.2% and 92.3%; 60.0%, 95.7%, 75.0% and 91.7%; 70.0%, 63.0%, 29.2% and 90.6%, respectively. IVST has the highest accuracy in predicting LBBaP failure; the thicker the LVST, the lower the success rate of LBBaP.

### 3.5. Cardiovascular Pharmacotherapy

Compared with baseline values, the number of patients taking diuretic agents and digoxin significantly decreased in LBBaP groups (all *p* < 0.001). However, there were no significant differences in the numbers of patients taking angiotensin-converting enzyme inhibitors (ACEI), angiotensin receptor blockers (ARB), angiotensin receptor neprilysin inhibitor (ARNI), and β-blockers (*p* = 0.482 and *p* = 0.388) (Table 6).

### 3.6. Adverse Events

All patients had at least one hospitalization for HF within 1 year before LBBaP, but only two patients (4.3%) had HF-related hospitalization within 12 months of LBBaP. Both were induced by severe infection. In the LVSP group, one patient with cardiomyopathy was rehospitalization for HF during the 1-year follow-up period. There were no records of patient deaths during the follow-up period in both LBBaP and LVSP groups.

## 4. Discussion

The main findings of this retrospective, observational study are as follows: (1) At the time of implantation and 1-month and 1-year follow-up, QRSd were longer than that of the LBBaP group during the same period (all *p* < 0.001). The pacemaker parameters remained stable in both the LBBaP and LVSP groups, and no severe complications occurred at implantation or during the follow-up period. (2) At 1-month and 1-year follow-up after LBBaP and AVNA, the LVEF, LVEDd, and NYHA classifications continued to improve (all *p* < 0.05). Greater numerical improvements were observed in LVEF, LVEDd, and LAAPD at LBBaP group. Additionally, a more significant improvement in NYHA classification was observed in LBBaP group at 1-year follow-up when compared with the LVSP group (*p* = 0.021). (3) Baseline LVEF and QRSd change at implantation can predict the magnitude of improvement of LVEF at 1-year after LBBaP and AVNA. The lower the baseline LVEF, the smaller the QRSd broadening, or the more obvious the QRSd shortening at implantation, the more significant the improvement of LVEF at 1-year follow-up. (4) Baseline RALRD, the degree of TR, and IVST may be the factors affecting the success of LBBaP. (5) Compared with baseline values, the number of patients taking diuretic agents and digoxin significantly decreased after LBBaP and AVNA (all *p* < 0.001). 

His-Purkinje system pacing (including HBP and LBBaP) can restore the intrinsic electromechanical activation sequence of the heart, avoiding ventricular dyssynchrony and improving cardiac function. Previous studies confirmed the safety, efficacy, and clinical applications of HBP. However, it must be mentioned that HBP also has its insufficiencies, such as both undersensing and oversensing issues, having a higher pacing threshold owing to the fibrous structure (less myocardium), and having an increased battery drain because of inherently higher pacing thresholds [10,11,12,13]. Concurrent AVNA presents even further challenges given the close proximity of the AVN and optimal HBP pacing site. The pacing area of LBBaP is supposed to be the LBB area, including the trunk or left anterior and posterior fascicle of the LBB. LBB is a diffuse fanlike structure broadly distributed over the left septal surface. The anatomical characteristics of LBB provide a wider target for pacing, which makes LBBaP easier to perform than HBP. In addition, the left bundle branch area is not enclosed by fibrous sheaths similar to those around the His bundle. Therefore, the pacemaker parameters of LBBaP were more satisfactory and stable than those in HBP. LBBaP delivers physiological pacing to achieve electrical and mechanical synchrony of the LV. LBBaP captures the distal part of the conduction system and can more easily cross the block site. LBBaP and HBP can produce similar electrical and mechanical synchrony, and LBBaP and HBP showed similar improvements in symptoms and LV function [8,16,17].

The applications of LBBaP in symptomatic bradycardia, HF and AF have achieved good results preliminarily, while there are still few studies on permanent LBBaP combine with AVNA for treating AF patients with symptomatic HF. The latest research shows that AVNA in the presence of an LBBaP lead is associated with a higher success rate and fewer acute and chronic lead-related complications; LBBaP preserves left ventricular systolic function in patients with refractory AF post AVNA [18]. For patients with AF and HF, our study shows that LBBaP in combination with AVNA is safe and feasible. The patient’s clinical symptoms and cardiac function improved significantly during the follow-up period. Additionally, the pacemaker parameters remained stable and no severe complications occurred at implantation or during the follow-up period. The results are in line with previous studies.

However, in our study, there are still ten patients who failed in five attempts of lead positioning and finally received permanent LVSP. We further explored the factors affecting the success of LBBaP. An analysis on the baseline heart structure of the two groups found that baseline IVST, the degree of TR, and RALRD may be the factors related to the success of LBBaP. The LBBaP procedure rotated the 3830 lead into the interventricular septum and allowed it to reach the left ventricular surface LBB area, directly pacing the conduction system and rapidly agitating the entire ventricle to achieve electro-mechanical synchronization. (1) When the interventricular septum was thickened, it was difficult to rotate the 3830 lead deep into the interventricular septum to reach the left ventricular endocardial surface and capture the left bundle branch conduction system. Additionally, septal fibrosis existed in some patients with thickened interventricular septum and further increased the difficulty of screwing the 3830 lead into the deep ventricular septum. (2) Large right atrium might lead to cardiac rotation and insufficient sheath support. C315His sheath floating in the large right atrium increased the difficulty of crossing the tricuspid annulus. Even if the C315His sheath crossed the tricuspid annulus successfully, it was difficult for the sheath to contact the ventricular myocardium due to the insufficient length of the sheath entering in the ventricle [19]. (3) Severe TR promoted the enlargement of the right atrium diameter. Meanwhile, the backflow of blood from the right ventricle into the right atrium during systole impacted the sheath and 3830 lead, which significantly increased the difficulty of lead implantation. Clinically, it is very necessary to comprehensively evaluate the cardiac structure and cardiac function of patients before operation. For patients with huge cardiac chamber, heart transposition, horizontal or vertical heart, or transposition of the great arteries, “shaping the C315His sheath” or using “sheath in sheath (nested coronary sinus sheath outside C315His sheath)” to improve maneuverability and provide the strong backup-force for device delivery [8]. 

Additionally, we found that the pacing QRSd and stim-LVAT of LBBaP group were significantly shorter than those of LVSP group. Pacing QRSd can reflect the synchronization of left and right ventricular contraction to a certain extent. Stim-LVAT is a suitable subrogate indicator of evaluating left ventricular activation. LBBAP is a physiological pacing approach characterized as direct capture of the main left bundle or each of the fascicle branches. After pacing the LBB area, there was a narrow QRSd and stim-LVAT, indirectly indicating that the left and right ventricular synchronization was good. LVSP activated the ventricular myocardium and not the specialized conduction system; therefore, it does not achieve complete electrical resynchronization. The pacing QRSd and Stim-LVAT were longer. Long-term alteration of ventricular activation causes persistent electrical remodeling via a mechano-electrical feedback mechanism. This further induces significant changes in ventricular structure and function, leading to ventricular remodeling and HF. In addition, previous studies found that the selection of an optimal pacing site during operation mainly depends on imaging. Therefore, the pacing lead of some patients may not be located on the left side of the septum, which may further decrease the benefit of LVSP [20].

## 5. Limitation

(1) There is still a lack of long-term, large-sample, and multiple-centered randomized controlled prospective trials to confirm our observation further. (2) The sample size in our study was relatively small, which might have led to statistical bias. (3) We still lack a high level of evidence supporting whether LVSP combined with AVNA can benefit AF patients with HF and whether LBBaP is superior to LVSP. (4) This study did not investigate whether beneficial clinical outcomes by LBBaP combined with AVN ablation in patients with AF and HF would be better than those from other pacing strategies (such as RVAP or BiVP-CRT).

## 6. Conclusions

LBBaP combined with AVNA is safe and effective for AF patients with HF. The cardiac function was significantly improved, and the pacemaker parameters remained stable. No serious adverse events occurred during the follow-up period. Baseline RALRD, the degree of TR, and IVSP may be the factors affecting the success of LBBaP.

## Figures and Tables

**Table 1:** Baseline characteristics (number (%), $\overset{ˉ}{x}\pm S$).

Baseline Characteristics	LBBaP + AVNA (*n* = 46)	LVSP + AVNA (*n* = 10)	*p*-Value
Age, years	75.5 ± 8.0	74.6 ± 7.9	0.748
Male	24 (52.2%)	6 (60.0%)	0.920
BMI, kg/m^2^	23.9 ± 3.7	25.7 ± 2.6	0.151
Smoking	9 (19.6%)	3 (30.0%)	0.761
Alcoholism	8 (17.4%)	2 (20.0%)	1.000
Hypertension	32 (69.6%)	5 (50.0%)	0.415
Diabetes	12 (26.1%)	2 (20.0%)	1.000
Coronary heart disease	7 (15.2%)	1 (10.0%)	1.000
PCI history	5 (10.9%)	1 (10.0%)	1.000
Myocardial infarction	2 (4.3%)	0 (0)	1.000
Myocardiopathy	1 (2.7%)	4 (40.0%)	0.003
Stroke history	6 (13.0%)	2 (20.0%)	0.943
Kidney dysfunction	2 (4.3%)	1 (10.0%)	0.452
Cancer	3 (6.5%)	0 (0)	1.000

BMI = body mass index, PCI = percutaneous coronary intervention, LBBaP = left bundle branch area pacing, LVSP = left ventricular septal pacing, AVNA = atrioventricular node ablation.

**Table 2:** Comparison of baseline and pacing heart rates, QRSd and stim-LVAT (($\overset{ˉ}{x}\pm S$); median (IQR, 25–75%)).

Parameters	LBBaP + AVNA (*n* = 46)	LVSP + AVNA (*n* = 10)	*p*-Value
Heart rate, beats/min			
Baseline heart rate	92.0 ± 25.7	94.9 ± 24.5	0.743
Pacing heart rate	70.0 ± 0	70.0 ± 0	1.000
QRSd, ms			
Baseline QRSd	89.0 (82.0, 100.0)	97.0 (89.0, 115.5)	0.092
At implantation	109.0 (105.0, 117.0) ^a^	124.0 (119.3, 127.8) ^a^	<0.001
1-month	110.0 (105.0, 115.3) ^a^	124.0 (120.0, 130.0) ^a^	<0.001
1-year	110.0 (102.0, 114.0) ^a^	127.5 (124.5, 131.3) ^a^	<0.001
Stim-LVAT, ms	73.5 (69.0, 77.0)	91.5 (83.8, 100.5)	<0.001

QRSd = QRS duration, Stim-LVAT = pacing stimulus to left ventricular activation time, LBBaP = left bundle branch area pacing, LVSP = left ventricular septal pacing, AVNA = atrioventricular node ablation. ^a^ = intragroup comparison, compared to baseline QRSd, *p* < 0.05.

**Table 3:** Comparison of pacemaker parameters (($\overset{ˉ}{x}\pm S$); median (IQR, 25–75%)).

Parameters	LBBaP + AVNA (*n* = 46)				LVSP + AVNN (*n* = 10)			
	At Implantation	1-Month	1-Year	*p*-Value	At Implantation	1-Month	1-Year	*p*-Value
Pacing threshold, V	0.80 (0.50, 1.00)	0.75 (0.50, 0.76)	0.75 (0.50, 0.75)	0.177 *	0.88 (0.50, 1.00)	0.75 (0.50, 1.00)	0.75 (0.50, 1.00)	0.509 *
R wave amplitude, mV	11.8 ± 3.7	11.9 ± 3.5	12.3 ± 3.4	0.529 *	11.9 ± 2.3	12.1 ± 3.0	12.3 ± 2.1	0.422 *
Pacing impedance, ohms	761.0 ± 191.0	603.1 ± 103.8	556.7 ± 103.7	<0.001 *	796.7 ± 126.2	605.2 ± 97.2	545.8 ± 76.5	<0.001 *
Pacing percentage, %	NA	99.0 (98.0, 99.8)	99.8 (98.3, 100.0)	0.430	NA	99.3 (98.8, 99.9)	99.4 (97.8, 100.0)	1.000

LBBaP = left bundle branch area pacing, LVSP = left ventricular septal pacing, AVNA = atrioventricular node ablation, NA = not applicable. * Repeated-measures analysis of variance.

**Table 4:** Comparison of NYHA classifications and echocardiographic parameters (($\overset{ˉ}{x}\pm S$); median (IQR, 25–75%)).

Parameters	LBBaP + AVNA (*n* = 46)				LVSP + AVNA (*n* = 10)			
	Baseline	1-Month	1-Year	* *p*-Value	Baseline	1-Month	1-Year	* *p*-Value
NYHA classification	3.0 (2.0, 3.3)	2.0 (2.0, 3.0) ^a^	2.0 (1.0, 2.0) ^bc^	<0.001	3.0 (2.0, 3.0)	3.0 (2.0, 3.0)	2.0 (2.0, 3.0) ^b^	0.005
Echo parameters								
LVEF, %	53.0 ± 11.9	56.7 ± 11.1 ^a^	60.4 ± 8.8 ^bc^	<0.001	53.2 ± 11.7	56.0 ± 10.8	57.4 ± 5.9	0.159
LVEDd, mm	49.5 ± 5.8	48.4 ± 5.7 ^a^	46.3 ± 5.6 ^bc^	<0.001	47.5 ± 4.8	48.1 ± 5.5	46.9 ± 6.6	0.450
LAAPD, mm	47.8 ± 7.3	47.1 ± 6.7	45.8 ± 3.8 ^bc^	0.004	52.1 ± 7.0	51.6 ± 7.0	51.1 ± 7.0	0.782
RALRD, mm	45.1 ± 6.6	44.7 ± 6.1	43.6 ± 6.9 ^b^	0.076	55.1 ± 11.0 ^d^	54.7 ± 11.5	51.6 ± 8.2	0.075
RASID, mm	59.0 ± 8.2	58.3 ± 7.5	57.7 ± 8.4	0.345	69.8 ± 16.5	70.2 ± 16.8	70.2 ± 16.2	0.772
RVLRD, mm	22.0 (20.0, 24.0)	22.0 (20.0, 24.0)	22.0 (20.0, 24.0)	0.586	24.0 (20.0, 30.3)	24.5 (20.8, 20.0)	25.5 (19.8, 31.3)	0.748
MR	1.0 (1.0, 2.0)	1.0 (1.0, 2.0)	1.0 (1.0, 2.0) ^b^	0.028	1.0 (1.0,3.0)	1 (1.0, 2.3)	1,5 (1.0, 2.0)	0.673
TR	1.0 (1.0, 2.0)	1.0 (1.0, 2.0)	1.0 (1.0, 2.0)	0.476	2.0 (1.0, 3.0) ^d^	2.0 (1.0, 2.3)	1.0 (1.0, 2.0)	0.594
IVST, mm	10.0 (10.0, 11.0)	11.0 (10.0, 11.0)	10.0 (10.0, 11.0)	0.172	14.5 (11.0, 16.3) ^d^	14.0 (11.0, 16.3)	14.5 (10.8, 16.5)	0.599

NYHA = New York Heart Association, LVEF = left ventricular ejection fraction, LVEDd = left ventricular end-diastolic diameter, LAAPD = left atrial anterior-posterior diameter, RALRD = right atrial left-right diameter, RASID = right atrial superior-inferior diameter, RVLRD = right ventricular left-right diameter, MR = mitral regurgitation, TR = tricuspid regurgitation, IVST = interventricular septum thickness, LBBaP = left bundle branch area pacing, LVSP = left ventricular septal pacing, AVNA = atrioventricular node ablation, Echo = echocardiography. ^a^ = intragroup comparison, comparison of baseline value and 1-month follow-up value, *p* < 0.05; ^b^ = intragroup comparison, comparison of baseline value and 1-year follow-up value, *p* < 0.05; ^c^ = intragroup comparison, comparison of 1-month and 1-year follow-up values, *p* < 0.05; ^d^ = compared with the baseline value of LBBaP group, *p* < 0.05. * Repeated-measures analysis of variance.

**Table 5:** The magnitude of improvement of NYHA classifications and echocardiographic parameters (median (IQR, 25–75%) $\overset{ˉ}{x}\pm S$).

Parameters	1-Month			1-Year		
	LLBBaP + AVNA (*n* = 46)	LVSP + AVNA (*n* = 10)	*p*-Value	LBBaP + AVNA (*n* = 46)	LVSP + AVNA (*n* = 10)	*p*-Value
ΔNYHA classification	−1.0 (−1.0, 0)	0 (−1.0, 0)	0.085	−1.0(−2.0, −1.0)	−0.5(−1.0, 0)	0.014
ΔLVEF, %	3.7 ± 7.0	2.8 ± 4.9	0.713	7.4 ± 9.4	4.2 ± 7.7	0.325
ΔLVEDd, mm	−1.2 ± 3.5	0.6 ± 2.6	0.140	−3.3 ± 4.4	−0.6 ± 2.8	0.074
ΔLAAPD, mm	−0.9 ± 3.3	−0.5 ± 4.4	0.744	−2.5 ± 4.7	−0.9 ± 4.9	0.377
ΔRALRD, mm	−0.4 ± 4.1	−0.4 ± 3.0	0.983	−1.5 ± 4.5	−3.5 ± 5.2	0.222
ΔRASID, mm	−0.6 ± 3.3	0.4 ± 5.4	0.455	−1.3 ± 5.6	0.4 ± 7.0	0.434
ΔRVLRD, mm	0 (−1.0, 1.0)	0 (−2, 1.3)	0.472	0 (−0.3, 1.3)	0.5 (−1.3, 1.3)	0.612
ΔMR	0 (0, 0)	0 (0, 0)	0.930	0 (−0.3, 0)	0 (−0.3, 0)	0.663
ΔTR	0 (0, 0)	0 (−0.3, 0)	0.742	0 (0, 0)	0 (−0.3, 0)	0.978
ΔIVST, mm	0 (0, 0.3)	0 (0, 0)	0.184	0 (−0.3, 0)	0 (−1.0, 0.3)	0.947

NYHA = New York Heart Association, LVEF = left ventricular ejection fraction, LVEDd = left ventricular end-diastolic diameter, LAAPD = left atrial anterior-posterior diameter, RALRD = right atrial left-right diameter, RASID = right atrial superior-inferior diameter, RVLRD = right ventricular left-right diameter, MR = mitral regurgitation, TR = tricuspid regurgitation, IVST = interventricular septum thickness, LBBaP = left bundle branch area pacing, LVSP = left ventricular septal pacing, AVNA = atrioventricular node ablation. Δ = the magnitude of improvements at 1-month or 1-year follow-up.

**Table 6:** Changes in cardiovascular pharmacotherapy (number).

Medicine	LBBaP + AVNA (*n* = 46)			LVSP + AVNA (*n* = 10)		
	Baseline	1-Year	*p*-Value	Baseline	1-Year	*p*-Value
Diuretics *	32	13	<0.001	9	5	0.141
β-Blockers	31	27	0.388	7	7	1.000
ACEI/ARB/ARNI	32	35	0.482	6	7	1.000
Digoxin	16	2	<0.001	3	1	0.582

ACEI = angiotensin-converting enzyme inhibitor, ARB = angiotensin receptor blocker, ARNI = angiotensin receptor neprilysin inhibitor, LBBaP = left bundle branch area pacing, LVSP = left ventricular septal pacing, AVNA = atrioventricular node ablation. * Diuretics = furosemide, torasemide, spironolactone.

## Data Availability

The data presented in this study are available on request from the corresponding author. The data are not publicly available due to privacy restrictions.
